# The minimally invasive open video-assisted approach in surgical thyroid diseases

**DOI:** 10.1186/1471-2482-5-9

**Published:** 2005-04-27

**Authors:** Massimo Ruggieri, Andrea Straniero, Alessandra Mascaro, Mariapia Genderini, Massimino D'Armiento, Patrizia Gargiulo, Angela Fumarola, Pierpaolo Trimboli

**Affiliations:** 1 Department of Surgical Sciences and Applied Medical Technologies "Francesco Durante", University of Rome "La Sapienza", Rome, Italy; 2 Department of Experimental Medicine and Pathology, Chair of Endocrinology, University of Rome "La Sapienza", Rome, Italy; 3 Department of Medicine, University of Rome "La Sapienza", Rome, Italy

**Keywords:** minimally invasive thyroidectomy, MIVA, video-assisted surgery

## Abstract

**Background:**

The targets of minimally invasive surgery (MIVA) could be summarised by: achievement of the same results as those obtained with traditional surgery, less trauma, better post-operative course, early discharge from hospital and improved cosmetic results. The minimally invasive techniques in thyroid surgery can be described as either endoscopic "pure" approach (completely closed approach with or without CO_2 _insufflation), or "open approach" with central neck mini-incision or "open video-assisted approach". Traditionally, open thyroidectomy requires a 6 to 8 cm, or bigger, transverse wound on the lower neck. The minimally invasive approach wound is much shorter (1.5 cm for small nodules, up to 2–3 cm for the largest ones, in respect of the exclusion criteria) upon the suprasternal notch.

Patients also experience much less pain after MIVA surgery than after conventional thyroidectomy. This is due to less dissection and destruction of tissues.

Pathologies treated are mainly nodular goiter; the only kind of thyroid cancer which may be approached with endoscopic surgery is a small differentiated carcinoma without lymph node involvement.

The patients were considered eligible for MIVA hemithyroidectomy and thyroidectomy on the basis of some criteria, such as gland volume and the kind of disease. In our experience we have chosen the minimally invasive open video-assisted approach of Miccoli et al. (2002). The aim of this work was to verify the suitability of the technique and the applicability in clinical practice.

**Methods:**

A completely gasless procedure was carried out through a 15–30 mm central incision about 20 mm above the sternal notch. Dissection was mainly performed under endoscopic vision using conventional endoscopic instruments. The video aided group included 11 patients. All patients were women with a average age of 54.

**Results:**

We performed thyroidectomy in 8 cases and hemithyroidectomy in 3 cases. The operative average time has been 170 minutes.

**Conclusion:**

Nowadays this minimally invasive surgery, in selected patients, clearly demonstrates excellent results regarding patient cure rate and comfort, with shorter hospital stay, reduced postoperative pain and most attractive cosmetic results.

## Background

The targets of minimally invasive surgery (MIVA) could be summarised by: achievement of the same results as those obtained with traditional surgery, less trauma, better post-operative course, early discharge from hospital and improved cosmetic results.

After the first endoscopic parathyroidectomy, performed and described by Gagner in 1996 [4], several surgeons reported their experiences with minimally invasive and video-assisted surgery of the neck [1-30]. Shimizu successfully treated more than 120 patients using the anterior neck lifting method (VANS) using the abdominal wall-lift method [6,7].

The minimally invasive techniques in thyroid surgery can be described as either endoscopic "pure approach" (completely closed approach with or without CO_2 _insufflation) [9,10,12,21,22,27,30], or "open approach" with central neck mini-incision [8]or "open video-assisted approach" [1-3,5,11,13-20,23-26,28,29]. Traditionally, open thyroidectomy requires a 6 to 8 cm, or bigger, transverse wound on the lower neck. The minimally invasive approach wound is much shorter (1.5 cm for small nodules, maximum 2–3 cm for the largest, in respect of the exclusion criteria) upon the suprasternal notch.

In many papers, Miccoli et al. confirm the suitability of the minimally invasive video-assisted (MIVA) approach in performing hemithyroidectomy and thyroidectomy [12-18]. In their procedure a 15–20 mm transversal skin incision was made 2 cm above the sternal notch.

In our experience we have chosen the minimally invasive open video-assisted approach of Miccoli et al. (2002). The aim of this work was to verify the suitability of the technique and the applicability in clinical practice.

## Methods

### Patients

In our first year of this study 11 patients were selected for video-assisted surgery. The patients selected were 11 female with an average age of 54 (range 32 to 78). Preoperative evalutation (biochemical assessment, ultrasonography, and fine needle aspiration biopsy, in some cases) was obtained in all cases. Preoperative diagnosis was multinodular goiter in 8 cases, toxic adenoma in 1 case and papillary carcinoma in 2 cases. Approximate thyroid volume was 12.6 ml.

Inclusion criteria are: (table 1)

**Table 1:** 

*Inclusion criteria*
single nodule or small goiter (toxic or not) of surgical competence
cranio-caudal axis of the lobes must not exceed 7 centimetres
largest transversal diameter of the nodule must not exceed 3,5 centimetres
total thyroid volume <15–25 ml
small (max 2 cm) differentiated carcinoma without lymph node involvement

Exclusion criteria are: (table 2)

**Table 2:** 

*Exclusion criteria*	
*Absolute*	*Relative*

previous neck surgery; big goiter; local advanced cancer; lymph node metastasis; medullary or undifferentiated carcinoma	Previous neck radiation therapy; Basedow disease; cronic thyroiditis.

### Surgical instruments

The instruments necessary for this kind of surgery are in part the same in use for the traditional one; however, this technique used, in particular, proper tools characterized by small diameter (max 2 mm) that could be also used in endoscopic vision: atraumatic spatulas, spatula-shaped aspirator, forceps and scissors. Nevertheless, for minimally invasive thyroidectomy, the primary instruments are the 30-degree 5-mm endoscope and the 14 cm-long Harmonic Scalpel Scissors (Ethicon ENDO-SURGERY, Inc.).

### Surgical procedures

**Figure 1:**
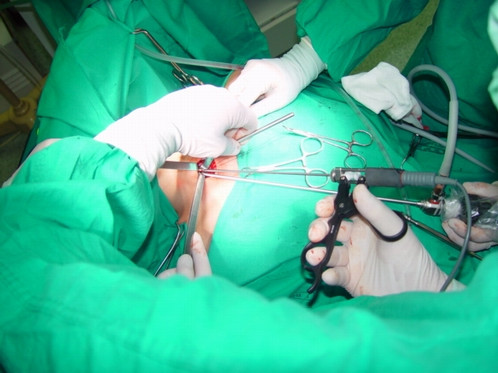
Minimally invasive video-assisted thyroidectomy. Two small retractors are used to maintain the operative space. The endoscope and the instruments are all inserted through the single single skin incision (an intraoperative view).

The neck is quite hyperextended. The surgical team consists of the surgeon and two assistants, one of whom must hold the camera. A 25–30 mm skin incision is performed about 2 cm above the sternal notch, in the middle line. The cervical linea alba is then opened as much as possible, making sure to avoid any minimal bleeding. The thyroid lobe on the affected side is then carefully dissected from the muscles. Two small retractors are used to medially retract and lift the thyroid and to laterally retract the muscles to maintain the operative space. A 30-degree 5-mm endoscope is inserted through the skin incision (Fig. 1).

Dissection of the thyrotracheal groove is completed under endoscopic vision by using small instruments.

**Figure 2:**
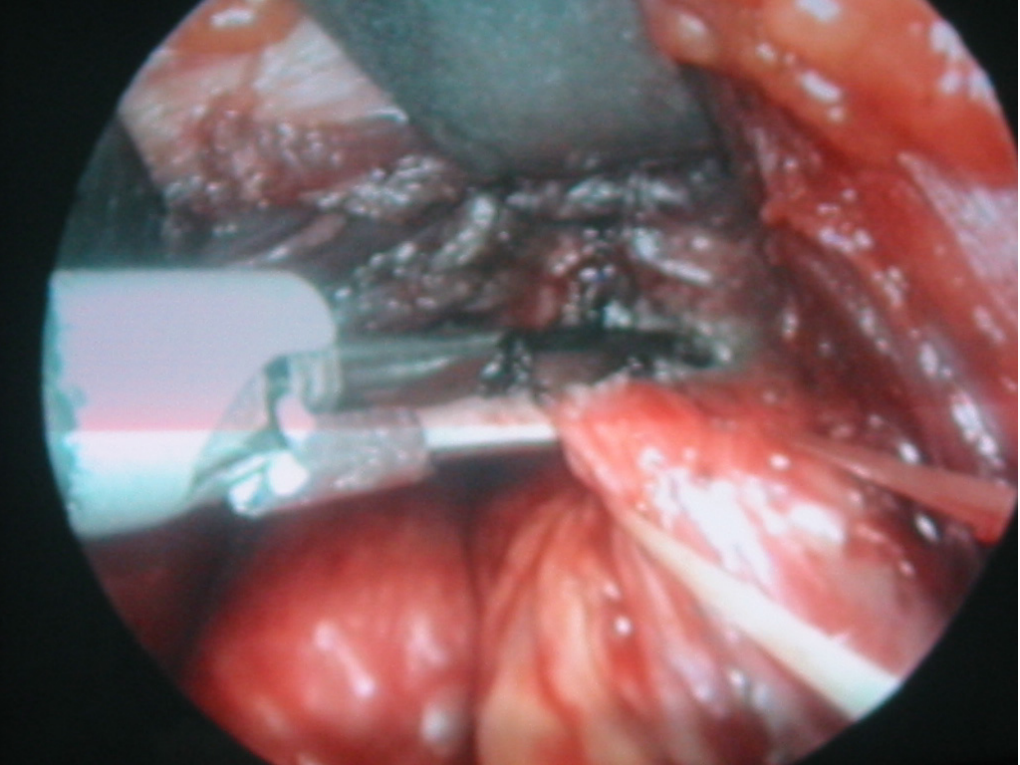
Minimally invasive video-assisted thyroidectomy. Upper pedicle sectioned by Harmonic Scalpel. Scissor (an endoscopic view).

The area must be completely bloodless, because even minimal bleeding makes the operation more difficult or impossible. To achieve haemostasis, we use small (3 mm) clips or the 5 mm, 14 cm-long Harmonic Scalpel scissors. The first vessel to be cut is the middle vein, if present, or the small veins between the jugular vein and the thyroid capsule. The spatula is used to separate the larynx from the vessels and to retract them laterally. The external branch of the superior laryngeal nerve can be easily identified during most procedures, once the different components of the upper pedicle have been prepared. The upper pedicle is then exposed and selectively cut by Harmonic Scalpel Scissors (Fig. 2).

The inferior vessels are also clipped and cut off, exposing the antero-lateral side of the trachea. The recurrent laryngeal nerve generally lies in the thyrotracheal groove, behind the Zuckerkandl tubercle. The recurrent nerve and the parathyroid glands are dissected and freed from the thyroid – these structures are well visualized by virtue of endoscope magnification [3]. Now the operation is conducted as in open surgery: the lobe is freed from the trachea, the isthmus dissected from the trachea and divided by harmonic scalpel. Finally the lobe is removed by conventional open technique. For total thyroidectomy, the same technique is repeated in the controlateral side. The muscles incision is sutured with reabsorbable suture and the wound is closed by intradermic adsorbable suture. We use drainage tubes (3.3 mm) that are introduced laterally.

## Results

The video assisted group included a total of 11 patients.

We performed in 8 cases a total thyroidectomy and in 3 an hemithyroidectomy. Operative average time was 170 minutes. In one case conversion to the traditional approach has been necessary for difficulties in finding the recurrent laryngeal nerve. No complications have happened, except from 2 cases of transitory hoarseness at the beginning of our experience. We have established, in our survey, that the length of the wound must not exceed 3 cm. This is because, untill this value, dissection of subplatysmal plane is not necessary, so avoiding postoperative pain or anterior neck discomfort. However the limit of 3 cm is largely conditioned by the experience of the operator and the duration of the intervention as well.

We obtained excellent results about patient cure rate and comfort, with short hospital stay, few postoperative pain and attractive cosmetic results.

## Discussion

Thyroid diseases primarily occurs from young to middle-age women who usually pay much attention to cosmetic results after thyroid surgery.

**Figure 3:**
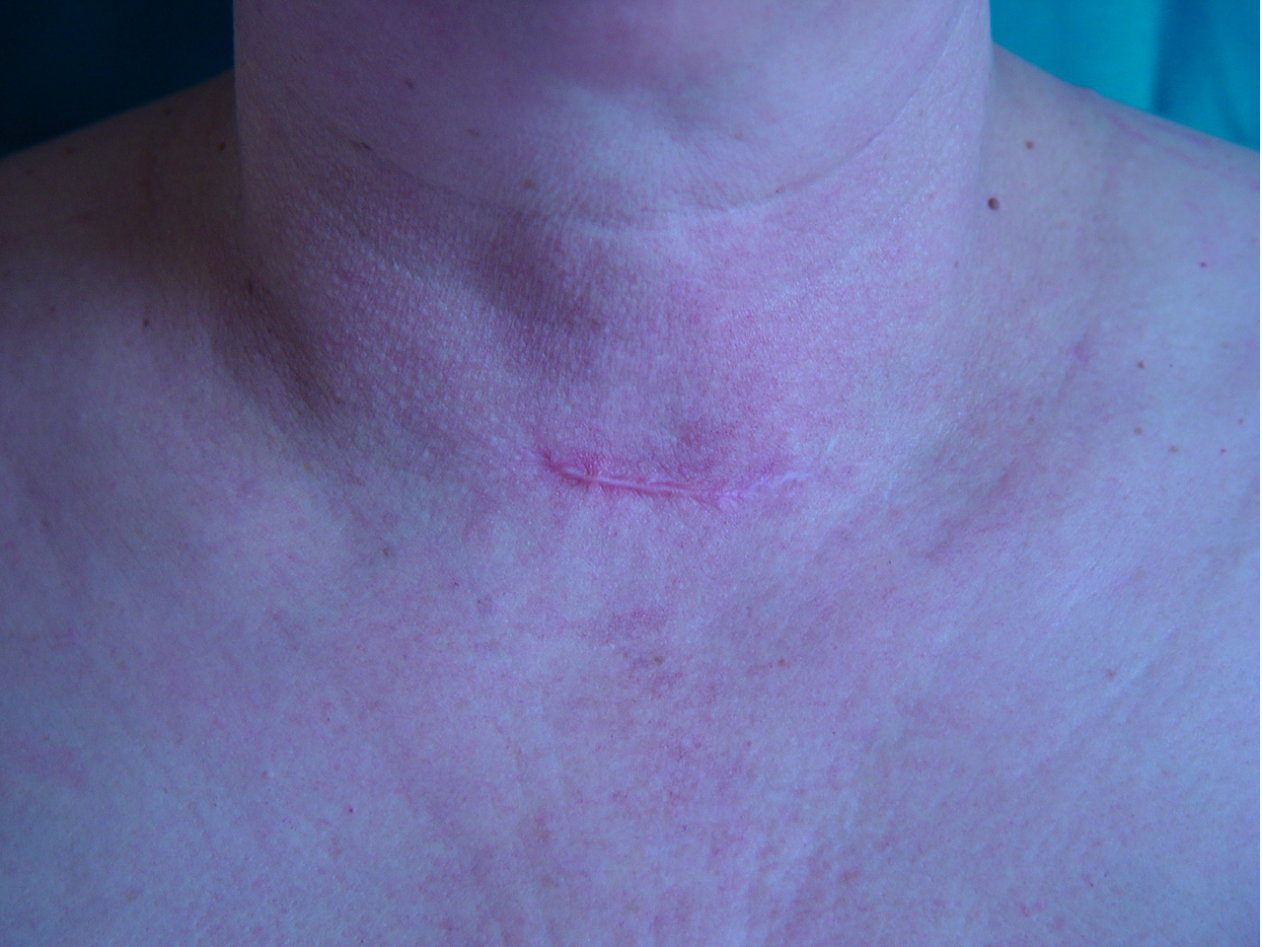
Minimally invasive video-assisted thyroidectomy. Using video-assisted endoscopic technique, the neck scar is only 1,5-maximum 3 cm in length on suprasternal notch, easily covered by a shirt.

Postoperative pain and recovery, following MIVA surgery, are shorter than those with conventional thyroidectomy, because there are fewer dissection and destruction of tissues and the dividing platysma doesn't complitely perform. Another aspect is the smaller number of cases of neck paresthesia (in the wound region) in the days following the operation. Of utmost importance, the minimally invasive approach wound is very short (1–2 cm for small nodules, up to 2–3 cm for the biggest, in respect of the exclusion criteria) upon the suprasternal notch, and is easily covered by a shirt (Fig. 3).

However, the development of a new surgical technique that minimizes the wound size is already possible, but the learning period is very long and the surgical tecnique is very hard. The operation time for minimally invasive video-assisted thyroidectomy is becoming comparable with that of conventional open technique [17,20,30].

It remains unclear whether the MIVA-thyroidectomy is suitable for the management of thyroid carcinoma or not. It is not yet recommended to use minimally invasive video-assisted thyroidectomy to manage thyroid malignancy until this surgical technique is mature enough to confidently dissect lymph nodes along the carotid sheath. The only kind of thyroid cancer that may be treated with endoscopic surgery is a small differentiated carcinoma without lymph node involvement [13].

Of course, minimally invasive video-assisted thyroidectomy has its limitations. MIVA-thyroidectomy is not suitable for repeated thyroid surgery because adhesions might interfere with the access of endoscope into the plane of the thyroid. Thyroid size is an important factor determining how difficult MIVA-approach would be, because the working space provided by the technique is limited. At this time, the maximum vertical axis of the lobes must not exceed 7 cm and the largest transversal diameter of the nodule must not exceed 3.5 cm. The total thyroid volume must not exceed 15–20 ml, echographically determined. It's not recommended to perform MIVA technique for goiter larger this size. Though in literature minimally invasive video-assisted approaches on thyroids well over 25 ml of volume and nodules greater than 3,5 cm are reported, at the moment it is not considered wise to operate lesions of such dimensions. The last limitation is the presence of a thyroidits diagnosed by biochemical or echographic signs [13,19]. As for the anterior neck lifting method (VANS) [6,7], we agree that it avoids the possibility of complications from carbon dioxide insufflation, but we would rather create the working space using two conventional retractors.

Complications of traditional thyroidectomy and MIVA are not different.

Transient recurrent nerve palsy and transient hypocalcemia are the more frequent, but the rate of these complications follows the learning curve. The operative time was about 80 minutes for a lobectomy and 130 minutes for a total thyroidectomy [12-18].

These operative times are slightly longer than those registered for conventional surgery in our Department, especially for the initial cases, because the development of a new technique always implies a learning period

Conversion to the traditional approach may be required in some cases for problems related to the bleeding from the vessels or the thyroid dimension.

A further issue of video-assisted techniques is a greater cost of this type of intervention, mainly due to the instruments that are required.

## Conclusion

This study demonstrates that MIVA thyroidectomy is a possible and safe procedure, when selection criteria are strictly followed. It can be considered a valid option because of its cosmetic advantages, which are particularly appreciated by young patients.

Follicular nodules, instead, are optimal candidates for this approach just because in Italy they are generally small. Besides, in patients affected by primary hyperparathyroidism due to a single adenoma and also presenting a thyroid nodule, this access allows treating both diseases with a single operation.

In our experience, the video-assisted surgery represents a remarkable improvment in the techniques of the surgery of the neck. Besides, a very important cosmetic improvment, especially for the patients turned to this surgery (young female patients in particular), this technique even reduces the invasivity of the surgical manoeuvres with a precocious reestablishment of the preoperatory well-being and with precocious de-hospitalization of the patients. Moreover, this method can be used nearly like routine surgery to facilitate the surgeon in preparation of the upper vascular pedicle, reducing the entity of the skin opening and in preparation of the superior miocutaneal edge, reducing the frequent paresthetic consequences ensuing.

This kind of technique can also be used in case of parathyroid glands disease, with less difficulties than thyroid surgery. In order to precisely define and clarify the role of minimally invasive video-assisted thyroidectomy or parathyroidectomy in the management of patients with thyroid and parathyroidectomy disease, larger studies and longer follow-up are requested. At present this kind of surgery clearly demonstrates excellent results regarding patient cure rate and comfort, with shorter hospital stay, fewer postoperative pain and attractive cosmetic results. In the future a way to optimise the benefits, might be the combination of the minimally invasive video-assisted surgery with minimal-aggressive anaesthesia, such as locoregional anaesthesia jointly with intravenous sedation [19].

## Pre-publication history

The pre-publication history for this paper can be accessed here:


